# Socio‐eco‐evolutionary dynamics in cities

**DOI:** 10.1111/eva.13065

**Published:** 2020-08-19

**Authors:** Simone Des Roches, Kristien I. Brans, Max R. Lambert, L. Ruth Rivkin, Amy Marie Savage, Christopher J. Schell, Cristian Correa, Luc De Meester, Sarah E. Diamond, Nancy B. Grimm, Nyeema C. Harris, Lynn Govaert, Andrew P. Hendry, Marc T. J. Johnson, Jason Munshi‐South, Eric P. Palkovacs, Marta Szulkin, Mark C. Urban, Brian C. Verrelli, Marina Alberti

**Affiliations:** ^1^ Department of Urban Design and Planning University of Washington Seattle WA USA; ^2^ Department of Biology Laboratory of Aquatic Ecology, Evolution and Conservation KU Leuven Leuven Belgium; ^3^ Department of Environmental Science, Policy, and Management University of California Berkeley CA USA; ^4^ Department of Ecology and Evolutionary Biology University of Toronto Toronto ON Canada; ^5^ Department of Biology University of Toronto Mississauga Mississauga ON Canada; ^6^ Centre for Urban Environments University of Toronto Mississauga Mississauga ON Canada; ^7^ Department of Biology Center for Computational and Integrative Biology Rutgers University Camden NJ USA; ^8^ School of Interdisciplinary Arts and Sciences University of Washington Tacoma Tacoma WA USA; ^9^ Facultad de Ciencias Forestales y Recursos Naturales Instituto de Conservación Biodiversidad y Territorio Universidad Austral de Chile Valdivia Chile; ^10^ Centro de Humedales Río Cruces Universidad Austral de Chile Valdivia Chile; ^11^ Institute of Biology Freie Universität Berlin Germany; ^12^ Leibniz Institut für Gewasserökologie und Binnenfischerei Berlin Germany; ^13^ Department of Biology Case Western Reserve University Cleveland OH USA; ^14^ School of Life Sciences Arizona State University Tempe AZ USA; ^15^ Applied Wildlife Ecology Lab, Ecology and Evolutionary Biology University of Michigan Ann Arbor MI USA; ^16^ Department of Evolutionary Biology and Environmental Studies University of Zurich Zurich Switzerland; ^17^ Department of Aquatic Ecology Swiss Federal Institute of Aquatic Science and Technology Duebendorf Switzerland; ^18^ Department of Biology Redpath Museum McGill University Montreal QC Canada; ^19^ Department of Biological Sciences and Louis Calder Center Fordham University Armonk NY USA; ^20^ Department of Ecology & Evolutionary Biology University of California Santa Cruz CA USA; ^21^ Centre of New Technologies University of Warsaw Warsaw Poland; ^22^ Center of Biological Risk and Department of Ecology and Evolutionary Biology University of Connecticut Storrs CT USA; ^23^ Center for Life Sciences Education Virginia Commonwealth University Richmond VA USA

**Keywords:** adaptation, anthropogenic, coupled human–natural systems, eco‐evo, socio‐ecological systems, urbanization

## Abstract

Cities are uniquely complex systems regulated by interactions and feedbacks between nature and human society. Characteristics of human society—including culture, economics, technology and politics—underlie social patterns and activity, creating a heterogeneous environment that can influence and be influenced by both ecological and evolutionary processes. Increasing research on urban ecology and evolutionary biology has coincided with growing interest in eco‐evolutionary dynamics, which encompasses the interactions and reciprocal feedbacks between evolution and ecology. Research on both urban evolutionary biology and eco‐evolutionary dynamics frequently focuses on contemporary evolution of species that have potentially substantial ecological—and even social—significance. Still, little work fully integrates urban evolutionary biology and eco‐evolutionary dynamics, and rarely do researchers in either of these fields fully consider the role of human social patterns and processes. Because cities are fundamentally regulated by human activities, are inherently interconnected and are frequently undergoing social and economic transformation, they represent an opportunity for ecologists and evolutionary biologists to study urban “socio‐eco‐evolutionary dynamics.” Through this new framework, we encourage researchers of urban ecology and evolution to fully integrate human social drivers and feedbacks to increase understanding and conservation of ecosystems, their functions and their contributions to people within and outside cities.

## 1 INTRODUCTION

**FIGURE 1:**
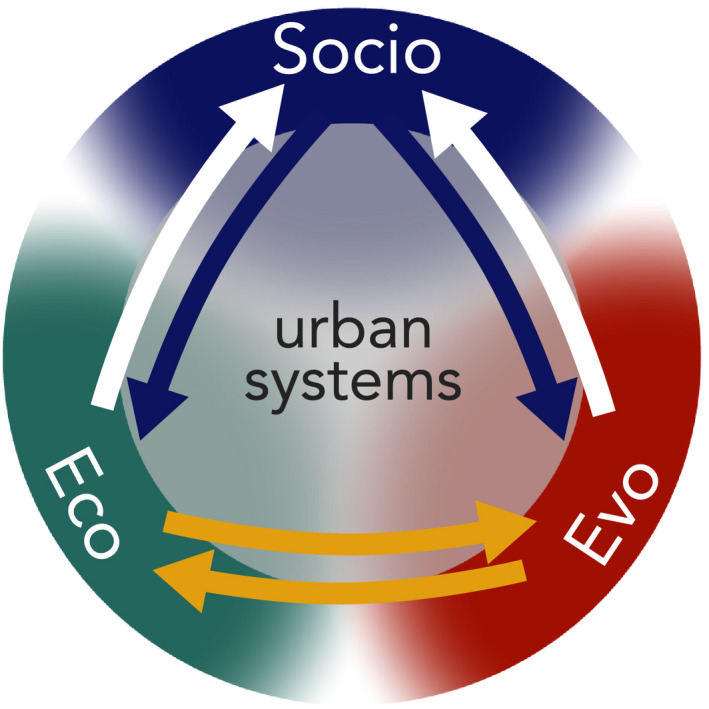
Urban ecosystems provide an opportunity to study contemporary evolution and ecological change inherent in eco‐evolutionary dynamics (yellow arrows). Eco‐evolutionary dynamics in urban ecosystems are strongly linked to human society. Characteristics of human society likely drive (blue arrows) and are impacted by (white arrows) ecological and evolutionary change

Humans construct and modify their surroundings to support the demands and desires of society (O’Brien & Laland, 2012). This phenomenon is particularly evident in cities, which are currently home to over half of the human population, a percentage predicted to rise to 66% by 2050 (UN, 2018). Urban expansion is rapid, with the global city footprint projected to double between 2015 and 2050, largely due to increased urban and suburban sprawl (Barrington‐Leigh & Millard‐Ball, 2020; Huang, Li, Liu, & Seto, 2019; Liu et al., 2020). As urban areas expand, they are becoming more socially heterogeneous, reflecting an influx of diverse people who bring myriad cultures from around the world (Qadeer, 1997, 2000; Sandercock, 1998). While humans and social processes are affecting all the planet’s biomes (Ellis, 2015), it is in urban ecosystems that human density and built habitats are the most pronounced. Cities have thus become representative of an urban “anthrobiome”—a set of ecosystems created and transformed by the people and societies that inhabit and depend on them (Alberti, 2008; Grimm et al., 2008; Pickett et al., 2001). Cities are unlike any other ecosystems because they are quintessentially built by and for one species: humans—a highly social, interconnected and omnipresent ecosystem engineer (Smith, 2007). As a result, the study of urban ecosystems should involve novel approaches by urban ecologists and evolutionary biologists to better integrate human social patterns and processes and build a truly synthetic understanding of the evolutionary ecology of cities (Figure 1).

Urban ecosystems (Definition: Box 1) are abiotically and biotically distinct from nonurban areas in that they feature human‐built structures, a high proportion of impervious surface, reduced vegetation cover, elevated pollution levels, and a disproportionately large number of exotic species (Grimm et al., 2008; Seto, Sánchez‐Rodríguez, & Fragkias, 2010). They are further characterized by altered patterns of connectivity, resource availability, inter‐ and intraspecific interactions, temperature, and habitat structure (Groffman et al., 2014; Walsh et al., 2005). Unsurprisingly, research has shown that these urban drivers have substantial effects on both ecological and evolutionary processes (Alberti, 2016; Donihue & Lambert, 2015; Johnson & Munshi‐South, 2017; Szulkin, Munshi‐South, & Charmantier, 2020). In many cases, the biological community composition, population demographics (Parris, 2016), phenotypic traits (Merckx, Kaiser, & Van Dyck, 2018) and genetic makeup (Munshi‐South 2012) of urban organisms differ substantially from their nonurban counterparts.

BOX 1 **Definitions**

**Table:** 

Urban Ecosystem	An ecosystem whose biological and physical characteristics are primarily engineered, modified and constructed by humans. In urban ecosystems, human society influences the relationships among organisms and between organisms and the physical environment. Urban ecosystems are characteristic examples of Coupled Human and Natural Systems (CHANS; Box 2).
Human Society	A group of human beings inhabiting and interacting within a common region, sharing and participating in the same culture (Tischler, 2006) or self‐sufficient system that usually persists longer than the lifespan of its individual members (Aberle, Cohen, Davis, Levy, & Sutton, 1950)
Urban Ecology	The interdisciplinary study of organismal and ecosystem patterns and processes within and among cities and their relationships with human activities. Urban ecology has increasingly incorporated the study of ecological interactions with human society in cities through frameworks such as CHANS (Box 2)
Urban Evolutionary Biology	The study of how urban form and processes shape adaptive (via natural selection) and nonadaptive (via mutation, gene flow and genetic drift) evolutionary dynamics that occur within or because of cities
Eco‐evolutionary dynamics	The interactions and feedbacks between ecological and evolutionary processes; both the ecological variation that affects evolution *and* the feedbacks of evolutionary change on ecological processes. Ecological and evolutionary feedbacks typically centre on contemporary adaptive evolution of ecologically relevant traits that alter how organisms interact and function in their ecosystems, for example influencing their productivity, excretion or resource consumption (Hendry, 2017)
Socio‐eco‐evolutionary dynamics	A framework for the integration of social, ecological and evolutionary patterns and processes that explicitly features the interactions and feedbacks among human society, ecology and both adaptive and nonadaptive evolution. This framework incorporates human social characteristics, such as economics, culture and policy, into the study of eco‐evolutionary dynamics in urban ecosystems (Figures 1and2)
Adaptive evolution	The process by which natural selection acts on heritable phenotypic trait variation in a population leading to the increased survival and reproduction (fitness) of individuals with certain trait values.
Nonadaptive evolution	Evolutionary change that is not driven by natural selection, including chance mutation, neutral genetic drift (random changes in the frequency of alleles in a population that is more pronounced in small, isolated populations) and gene flow (the transfer of genetic information among populations due to migration of individuals, gametes, and other propagules.
Nature’s contributions to people (NCP)	The essential and often nonreplaceable material and assistance (i.e. food, energy, other resources), nonmaterial (i.e. cultural, educational, inspirational) and regulating services (i.e. habitat, climate and resource maintenance, hazard protection) provided by nature that benefit human existence and well‐being. The concept of NCP encompasses and extends the former *ecosystem services* (Díaz et al., 2018). Though the new NCP framework does not specifically allude to detrimental feedbacks on humans, authors have also acknowledged *ecosystem disservices*, particularly in urban ecosystems (Shackleton et al., 2016). Authors have also recognized *evosystem services*—benefits to humans resulting from evolutionary change (Faith et al., 2010, 2017; Rudman et al., 2017). The concept of NCP is central to socio‐eco‐evolutionary dynamics, as it describes the feedbacks from ecology and evolution towards human society (Figure 2)
Evolving metacommunity framework	A framework describing the spatial context of eco‐evolutionary dynamics that considers sets of local communities linked by the dispersal of multiple species (a metacommunity) and the change in species interactions with the environment and with each other via evolution. This framework integrates community ecology and evolution in local patches with regional dispersal and gene flow among regional patches to understand eco‐evolutionary interactions at multiple scales (Urban et al., 2008)

**FIGURE 2:**
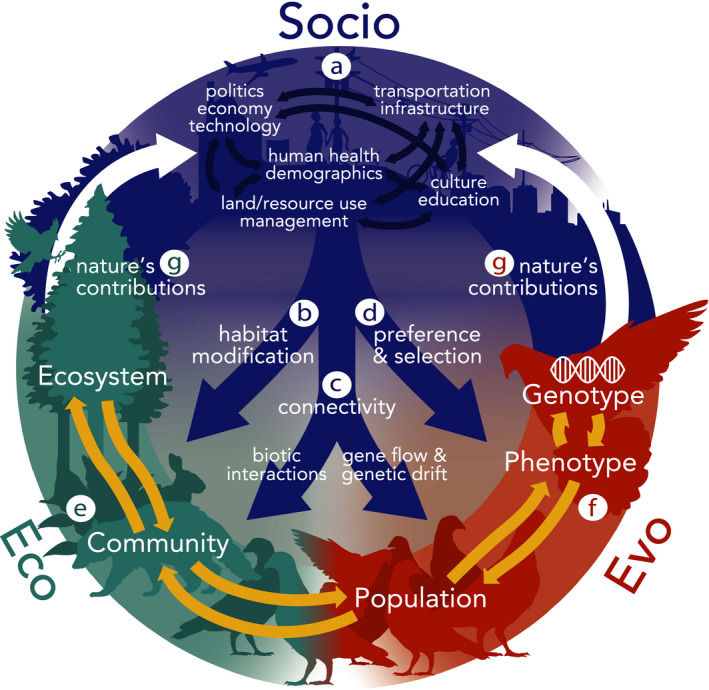
Detailed dynamics among social, ecological and evolutionary patterns and processes in urban systems. Social patterns and processes (a) encompass a diversity of political, economic, and technological drivers that are interrelated with transportation and infrastructure, culture and education, human population demographics, and land/resource use and management. Social drivers affect (b) ecology through habitat modification; (c) ecology (biotic interactions) and evolution (gene flow and genetic drift) through altering connectivity among habitats; and (d) ecology and evolution through selection for preferred genotypes and phenotypes. Ecological (e) and evolutionary (f) dynamics are linked through feedbacks between ecosystems, communities, populations, genotypes and phenotypes. Ecological and evolutionary feedbacks towards society take the form of nature’s contributions to people (g) including ecosystem services and disservices

The structure and composition of urban ecosystems are predominantly a consequence of human society (Definition: Box 1), which reflects the complex interplay among culture, economy, politics and technology (Avolio et al., 2018; Collins et al., 2000; Grove, Locke, & O’Neil‐Dunne, 2014; Marzluff, 2008). As a result, urban ecological and evolutionary processes are intrinsically influenced by social patterns and processes (Figure 2; Grove et al., 2014; Troy, Grove, & O’Neil‐Dunne, 2012). Not only are human activities an underlying driver of ecological and evolutionary processes in cities, these processes feed back to affect human health and well‐being through nature’s contributions to people (Definition: Box 1; Díaz et al., 2018), including both ecosystem (Daily, 1997) and “evosystem” (Faith et al., 2010; Faith et al., 2017; Rudman et al., 2017) services and disservices. These processes may further shape and reshape human attitudes and behaviours towards the environment and biodiversity conservation (Reddy et al., 2017).

As cities have grown, so too has interest in the myriad intersections between human life and the lives of other species. During the last three decades, the field of urban ecology (Definition: Box 1) has made large strides in integrating human social dimensions into the study of urban ecosystems by fostering new collaborations between natural and social scientists. These collaborations have uniquely explored how urbanization shapes ecological processes, promoting the understanding of cities as ecosystems where humans play a fundamental role in regulating environmental patterns and processes (Alberti, 2008; Liu et al., 2007). Studies on urban evolutionary biology (Definition: Box 1) have also increased in recent years (Johnson & Munshi‐South, 2017; Rivkin et al., 2019; Szulkin et al., 2020). Although some of the earliest work showing evidence of natural selection focused on urban adaptive evolution (Definition: Box 1; Kettlewell, 1958), recent advances in molecular techniques and a broader understanding of the role of gene flow and neutral evolution have contributed to a wealth of research on how nonadaptive evolution (Definition: Box 1)—including patterns of genetic drift and gene flow—operates in cities (Miles et al., 2019; Rivkin et al., 2019; Schmidt, Domaratzki, Kinnunen, Bowman, & Garroway, 2020; Szulkin et al., 2020). Increasing research on urban evolutionary biology has also coincided with the growing field of eco‐evolutionary dynamics (Definition: Box 1), which aims to understand the interactions and feedbacks between evolutionary and ecological processes (Fussmann, Loreau, & Abrams, 2007; Hendry, 2017; Schoener, 2011). Researchers of both urban evolutionary biology and eco‐evolutionary dynamics tend to focus on contemporary evolution in species that can have important ecological—or even social—feedbacks (Faith et al., 2010, 2017; Rudman et al., 2017); few, however, have examined the presence and strength of eco‐evolutionary dynamics in urban ecosystems (Alberti, 2015).

In recent years, interdisciplinary progress has been made showing how social processes influence ecological dynamics (Band et al., 2005; Liu et al., 2007), how evolutionary dynamics feed back on ecology (Fussmann et al., 2007; Hendry, 2017; Pelletier, Garant, & Hendry, 2009), and how evolutionary dynamics contribute to society (Faith et al., 2010; Palumbi, 2001). However, a general framework for addressing the relationships among all three dimensions—social, ecological and evolutionary—is still lacking. In particular, little research fully integrates urban evolutionary biology with eco‐evolutionary dynamics (but see Brans, Jansen, et al., 2017) and rarely do either of these fields fully consider the role of human social processes on the eco‐evolutionary dynamics in cities (but see Schell et al., 2020). We argue that cities present an opportunity to integrate the fields of social science, ecology, and evolutionary biology for the following reasons: (a) urban ecosystems are biotically and abiotically distinct, potentially resulting in unique effects on ecological and evolutionary dynamics compared to nonurban systems; (b) social patterns and processes are concentrated in cities, where they modify the ecological stage on which evolution takes place, thereby affecting urban eco‐evolutionary dynamics; (c) ecological and evolutionary processes in cities are likely to feed back on humans and society; and (d) these feedbacks might be magnified or dampened depending on the social and urban contexts in which they occur.

The goal of this perspective piece is to provide a “socio‐eco‐evolutionary dynamics” (Definition: Box 1) framework for evolutionary ecologists studying urban ecosystems. We highlight the importance of integrating social patterns, processes, and responses in research on urban ecology, evolutionary biology and eco‐evolutionary dynamics. Further, we use examples from specific study systems and describe how existing frameworks from research in these fields may be extended to include social dimensions. We close by laying the groundwork for future research on urban socio‐eco‐evolutionary dynamics with a set of empirical and theoretical guidelines and questions.

## 2 LINKING URBAN SOCIAL PROCESSES WITH ECOLOGY AND EVOLUTION

Characteristics of human society—demography, culture, governance, economics, and social organization (Odum, 1943; Tipps, 1973)—not only govern interactions among humans, but also influence human interactions with nature. Humans have always engaged in socio‐ecological and socio‐evolutionary relationships, whether through hunting and gathering, domestication and agriculture, or the use of natural resources to build civilizations and cities (Boivin et al., 2016; Sullivan, Bird, & Perry, 2017). Through these relationships, humans have not only fragmented and connected species’ populations, but also constructed and modified their ecological niches. A wealth of research from a diversity of disciplines (e.g. political ecology, cultural anthropology, sociology) has revealed the ubiquity of complex interactions between human society and nature through millennia and across geographic regions (Boivin et al., 2016; Ellis, 2015; O’Brien & Laland, 2012). This research has laid the groundwork for studying the interactions among social, ecological, and evolutionary dynamics in cities.

### 2.1 Social drivers of urban ecology

In recent years, urban ecology has emerged as a unified discipline, focusing on the many ways in which urbanization alters abiotic and biotic conditions that influence species interactions, patterns and processes and how they feed back to people via changes in ecosystem function (Collins et al., 2000; Grimm, Grove, Pickett, & Redman, 2000). Intraspecific (communication, mating behaviour, within‐species competition) and interspecific (mutualism, predation, herbivory, among‐species competition) interactions—including with humans—can differ significantly between urban and surrounding nonurban habitats (Miles, Breitbart, Wagner, & Johnson, 2019; Pereira‐Peixoto, Pufal, Staab, Feitosa Martins, & Klein, 2016; Rodewald, Shustack, & Jones, 2011). Urban ecology has increasingly integrated human social patterns and processes in the study of urban ecosystems (Alberti, 2008; Grimm et al., 2000; Marzluff, 2008; Tanner et al., 2014), recognizing that cities comprise a mosaic of natural and built habitats with varying disturbance across space and time (Pickett, Cadenasso, Childers, McDonnell, & Zhou, 2016; Savage, Hackett, Guénard, Youngsteadt, & Dunn, 2015).

Redefining cities as intrinsically coupled human and natural systems (also known as CHANS: Box 2) acknowledges not only that social decisions shape urban ecosystems, but also that ecological changes motivate important human decisions (Liu et al., 2007). Decisions and policies made at various social scales—individuals, neighbourhoods, businesses, or municipal and national governments—can both directly regulate and be regulated by urban decision‐making and its ecological effects (Pickett et al., 2016). For example, planted trees and gardens regulate air filtration and micro‐climates, sump ponds act as stormwater reservoirs, and restored soil and macrophyte communities treat sewage and chemical waste via nutrient uptake and bio‐ and phytoremediation (Jabeen, Ahmad, & Iqbal, 2009; Zipperer, Morse, & Gaither, 2011). Parks provide recreational and cultural amenities that not only benefit people and reshape ecological processes, but are fundamentally driven by human choices (Ackley, 2014; Bolund & Hunhammar, 1999; Leong, Bertone, Bayless, Dunn, & Trautwein, 2016). The CHANS (Box 2) literature has provided a useful framework for studying urban ecology, but it has yet to incorporate evolutionary biology and eco‐evolutionary dynamics.

BOX 2 **Coupled Human and Natural Systems (CHANS)**

Coupled human and natural systems (CHANS) are increasingly pervasive as human activities now influence most natural processes. Researchers recognize CHANS by explicitly acknowledging linked reciprocal interactions between humans and nature—often characterized by flows of material, energy, and information (Liu et al., 2007; McDonnell & Pickett, 1993). A critical, yet under‐recognized component of CHANS is their unexpected feedbacks. These include nonlinear responses and threshold conditions in which system components transition into alternative states, as well as time lags between a stressor and its effects and/or recognition of these effects and the subsequent decisions. Also characteristic of CHANS are emergent properties in which simultaneous changes across multiple variables produce new environmental contexts that cannot be adequately characterized by any single variable or be identified in the human or natural systems alone (Alberti et al., 2020). Given their complex and heterogeneous nature, cities typify CHANS. Urban ecologists have increasingly relied on the CHANS conceptual frameworks to understand human–nature connections and dynamics embedded within cities. Doing so has allowed urban ecologists to move from simply studying ecology that occurs within cities to understanding the ecology *of* cities (Grimm et al., 2008, 2000; Pickett et al., 2001). Cities are exemplary CHANS because they are characterized by substantial complexity in ecological, hydrological and geophysical structure and function across scales as well as complex social hierarchies—from individuals to households, neighbourhoods, municipalities, regions, and nations—with feedbacks occurring within and among various ecological and social scales (Grimm et al., 2008, 2000; Pickett et al., 2001). Because of this complexity, cities and their components cannot simply be understood by measuring human population sizes or densities and require a more comprehensive assessment of biophysical and social conditions.

### 2.2 Social drivers of urban evolution

A large body of research has revealed that the historical rise of aggregated human communities and subsequent origin of the first cities reflect deep interactions between social and evolutionary processes. The advent of the agrarian societies predating modern cities is reflected in the genomes of humans and domesticated species (O’Brien & Laland, 2012). For the past fifteen thousand years, cultural and agricultural practices have led to strong selection on numerous species (Driscoll, Macdonald, & O’Brien, 2009; Larson & Fuller, 2014) as well as coevolutionary relationships with humans (Jackson, 1996; Leach, 2003). For example, coevolution between humans and crop plants (Perry et al., 2007; Ye, Gao, Wang, Bar‐Yosef, & Keinan, 2017) and between humans and livestock (Tishkoff et al., 2007) is associated with the advent of agriculture and the abandonment of nomadic hunter–gatherer lifestyles. For example, genes for lactase that enable dairy consumption (Tishkoff et al., 2007), and amylase that aid starch consumption (Perry et al., 2007), show geographically spatial and cultural patterns of balancing selection for diverse diets.

Historical and contemporary evolutionary patterns in species most closely associated with humans can reflect social, cultural and even economic trends and trajectories. Indeed, biologists have learned a great deal about evolutionary processes through researching social‐evolutionary processes such as domestication. Darwin (1859) built his argument of evolution by natural selection through analogy with artificial selection in the domesticated rock pigeon (*Columba livia*) and other animals. Today, evidence suggests that some of the pigmentation patterns originally favoured by fancy pigeon breeders confer an adaptive advantage for urban pigeons (Vickrey et al., 2018), demonstrating the influence of past social preferences on the evolutionary history of a species. Domesticated dogs (*Canis familiarus*), which have undergone thousands of years of artificial selection, still commonly interbreed with wild coyote (*Canis latrans*; Mahan, Gipson, & Case, 1978) and wolf (*Canis lupus*; Pilot et al., 2018) populations. Studies have shown that dogs are often less likely to be neutered and more likely to be abandoned in lower‐income urban areas following widespread economic downturns (Morris & Steffler, 2011). Thus, the observed introgression of domestic dog alleles into nearby coyote or wolf populations could potentially be the result of socio‐economic patterns, though this has yet to be directly tested.

Some of the classic examples of adaptation by natural selection invoke urban social processes. Pollution and habitat degradation often accompany major technological innovations that are later followed by policies mitigating their damage. For example, during the industrial revolution in the United Kingdom, increasing urban activity deposited a layer of dark soot on the bark of surrounding trees that selected for rarer melanic variants of the commonly light‐coloured peppered moth (*Biston betularia*), which became more cryptic and less subject to predation (Cook & Saccheri, 2013; Hof et al., 2016; Kettlewell, 1958). The Clean Air Act, enacted in the UK in 1956, decreased pollutants, leading to an evolutionary reversal whereby light‐coloured moths again increased in frequency (Cook & Saccheri, 2013). In this iconic natural selection case study, the evolutionary trajectory of urban‐adjacent peppered moth populations ostensibly reflected human societal patterns of socio‐economic and technological innovations, their impacts, and environmental policy.

Today, many evolutionary biologists explore how species respond to novel selection pressures in urban environments (Alberti, 2015; Donihue & Lambert, 2015; Johnson & Munshi‐South, 2017; Szulkin et al., 2020). These selection pressures can vary over fine spatial and temporal scales (Donihue & Lambert, 2015), providing a more realistic context for studying in situ evolution. For example, populations of killifish (*Fundulus heteroclitus*) from four cities have convergently evolved novel adaptations which confer resistance to toxins in response to pollution in urban estuaries (Reid et al., 2016; Whitehead, Clark, Reid, Hahn, & Nacci, 2017). In another example, white clover (*Trifolium repens*) has shown repeated phenotypic convergence in the loss of cyanogenesis in response to urbanization (Case Study: Box 3a; Johnson et al., 2018; Santangelo, Johnson, & Ness, 2018; Thompson, Renaudin, & Johnson, 2016); there is also increasing evidence for adaptations to stressors such as urban heat islands (Brans & De Meester, 2018; Diamond, Chick, Perez, Strickler, & Martin, 2018), which are characteristics that are also reflective of income inequality among urban neighbourhoods (Chakraborty, Hsu, Manya, & Sheriff, 2019). Researchers have also shown that species might be insulated from selection pressures in urban environments that exclude their predators (Rebolo‐Ifrán, Tella, & Carrete, 2017), though little work has evaluated the evolutionary consequences of such relaxed pressures.

BOX 3 **Urban Socio‐Eco‐Evo Dynamics Case Studies**

(a) *Social determinants of rat ecology, evolution, disease transmission and pest management*

Brown or “Norway” rats (*Rattus norvegicus*) have coinhabited with humans for centuries by exploiting food and built structures (Byers et al., 2019; Gardner‐Santana et al., 2009). Brown rats show adaptive resistance to rodenticide commonly used in urban habitats (Desvars‐Larrive et al., 2017) and significant genetic differentiation at the city block scale where high‐traffic roadways limit gene flow across neighbourhoods (Combs, Byers, Himsworth, & Munshi‐South, 2019; Combs et al., 2018; Gardner‐Santana et al., 2009; Kajdacsi et al., 2013). Garbage management may also influence the population genetic structure of rats such that individuals in resource‐rich microhabitats are less likely to disperse and thus aggregate with more closely related kin within small areas (Gardner‐Santana et al., 2009). Unsecured food waste, dilapidated structures and overgrown vegetation all promote increases in rat infestation in urban areas (Murray et al., 2018; Walsh, 2014). In response to societal and economic neglect, low‐income communities often have the highest aggregation of attractants for brown rats (Byers et al., 2019; Kajdacsi et al., 2013; Murray et al., 2018; Peterson et al., 2020). These dynamics intrinsically link wealth inequality and rat urban ecology. Brown rats are notorious reservoirs of multiple zoonotic pathogens that have myriad negative health implications for humans (Gardner‐Santana et al., 2009; Kajdacsi et al., 2013; Richardson et al., 2017). Brown rats’ role as carriers of pathogens underscores the urgent public health priority for socio‐eco‐evo investigations that inform sustained and efficient pest management practices (Byers et al., 2019; Combs et al., 2019). Recent findings show how rats capitalize on urban centres and can thus inform pest management strategies (Combs et al., 2019). Disenfranchised communities with reduced infrastructure quality should feasibly receive the most targeted and sustained pest control efforts (Peterson et al., 2020). However, many of these communities are socially and economically neglected, receiving insufficient waste management and public services that would alleviate the conditions that attract brown rats. In combination, these studies demonstrate how social determinants shape ecological conditions that promote rat colonization and adaptation, resulting in negative feedbacks to society in one of the few, fully articulated examples of socio‐eco‐evolutionary dynamics in cities.
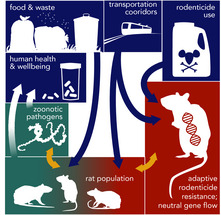

b) *Social landscape drivers and pesticides impact mosquito evolution and disease in cities*

Mosquitoes (including *Aedes aeqypti* and *Culpex pipiens)* are ubiquitous across the globe and are prominent vectors for human disease (e.g. Zika virus, malaria, dengue fever, West Nile virus; (Kalluri, Gilruth, Rogers, & Szczur, 2007; Rochlin, Turbow, Gomez, Ninivaggi, & Campbell, 2011). Pest management in cities is especially urgent because mosquitoes show accelerated larval growth and increased survivorship in urban environments due to greater densities of suitable breeding locations (small volumes of standing water), urban heat islands and reductions in predators due to insecticides and unsuitable habitat (Li, Dicke, Harvey, & Gols, 2014; Wilke et al., 2019). Insecticide application has also promoted resistance, and challenged pest management. Hence, mosquitoes generally tend to experience fitness benefits in cities, increasing the risk of pathogen transmission among humans (Kamdem et al., 2017; Medeiros‐Sousa, Fernandes, Ceretti‐Junior, Wilke, & Marrelli, 2017). Variation in urban infrastructure, driven by socio‐economics and urban planning, can be linked directly to the ecology and evolution of mosquito species. Low‐income cities and neighbourhoods have greater relative proportions of impervious surface cover, leading to more surfaces holding standing water (Ayala & Estrugo, 2014; Rochlin et al., 2011). Accordingly, impoverished neighbourhoods have larger mosquitoes in better condition, with increased survivorship and reproduction (Katz et al., 2019). Recent empirical work further shows that urban residents in low‐income neighbourhoods have greater risk of mosquito‐borne diseases, specifically West Nile virus in Washington, D.C., and Baltimore, Maryland (LaDeau, Leisnham, Biehler, & Bodner, 2013) and malaria in cities across sub‐Saharan Africa (De Silva & Marshall, 2012). Social drivers may additionally affect the rate of coevolutionary change between mosquito‐borne diseases (e.g. *Plasmodium*) and human resistance to those diseases (Ayala & Estrugo, 2014). For example, sickle cell anaemia, a disease characterized by malformed red blood cells, is typically lethal in people who inherit two copies of an allele with a mutation inhibiting haemoglobin production (Allison, 1954). However, heterozygotes (with just one sickle cell allele) have increased resistance to malaria, leading to the higher prevalence of the allele in urban, suburban and rural areas where malaria is common (Evans & Wellems, 2002). As countries in malaria‐affected areas continue to urbanize, the close coevolutionary association among humans, mosquitos and *Plasmodium* species may become an increasingly urban issue.
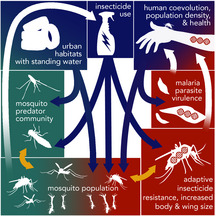

c) *Clover evolution, repeated loss of cyanogenesis and urban lawns*

The ecology and evolution of white clover (*Trifolium repens*), a perennial, herbaceous plant common in lawns and other human‐modified habitats, has been well studied in an urban context. Clover exhibits a Mendelian polymorphism for hydrogen cyanide production (cyanogenesis), which both defends against herbivores and reduces freezing tolerance. White clover repeatedly evolves decreased cyanogenesis in cities, due to putative selection from colder night‐time winter temperatures (Johnson et al., 2018; Santangelo et al., 2018; Thompson et al., 2016). White clover’s adaptations might in part lead to their high population densities in cities, where they feed back on the urban ecosystem and society. In particular, clover’s mutualistic rhizobial bacteria influence increase soil nitrogen (Hennig & Ghazoul, 2011) and its flowers provide a nectar resource for pollinators (Hicks et al., 2016; Larson et al., 2014; Theodorou et al., 2017). Still, despite its presence in many lawn seed mixes (Bormann, Balmori, & Geballe, 2011), white clover is often considered a weed and removed by homeowners, negatively affecting pollinator communities (Baude et al., 2016; Larson et al., 2014). Because of its strong association with humans, its importance for nutrient cycling and pollinators, and its evolution in cities, the urban white clover system presents an opportunity to study socio‐eco‐evolutionary dynamics. In particular, research could explore how land use and conversion, homeowner cultural habits, and household income predict clover presence in lawns and thus spatial heterogeneity in pollinator resource availability. If clover is removed, policies could encourage the planting of native species to support lost ecosystem functions.
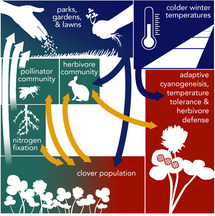

d) *Daphnia evolution, eutrophication, urban heat islands and trophic cascades*

*Daphnia* are common zooplankton species in urban, rural and natural freshwater ponds and lakes across the globe. They vary in several intraspecific life‐history, behavioural, and physiological traits that can elicit strong ecosystem‐level effects. *D. magna* show reduced body size, higher heat tolerance, faster pace of life, and altered stress physiology in urban populations compared to rural populations, which are most likely adaptations to warmer temperatures (Brans & De Meester, 2018; Brans, Jansen, et al., 2017). Smaller average body size in urban zooplankton communities that include *Daphnia* can have cascading effects on pond ecosystems (Gianuca, Pantel, & De Meester, 2016). While increased *Daphnia* thermal tolerance allows them to persist and suppress algae populations, smaller body size diminishes their capacity to do so (Gianuca et al., 2016). Reduced top‐down effects from primary consumers can result in the disappearance of emergent and submerged vegetation, eutrophication, and decline in amphibians, invertebrates and overall pond biodiversity (Blaustein et al., 2011; Huisman et al., 2018; Landsberg, 2002; Paerl & Otten, 2013). Algal blooms will likely increase with climate change and urbanization (Paerl & Huisman, 2009; Teurlincx et al., 2019; Waajen, Faassen, & Lürling, 2014) causing toxic conditions that are harmful for humans and pets (Huisman et al., 2018; Reid et al., 2019). Persistence of *D. magna* in urban and natural ponds is thus crucial for human health and well‐being. Yet, certain actions taken by humans can directly lead to their demise (Paerl & Huisman, 2009; Teurlincx et al., 2019; Waajen et al., 2014). For example, fertilizer run‐off and removal of submerged vegetation can result in anoxic conditions, fatal to *D. magna* and other zooplankton (Peretyatko, Teissier, De Backer, & Triest, 2009). Further, stocking of zooplanktivorous fish can reduce *Daphnia* abundance and thus their ability to control algae populations (Peretyatko et al., 2009). Shifts towards eutrophic pond ecosystems can negatively impact human psychological well‐being, hydrological balance, climate mitigation, nutrient retention, and bio‐ and phytoremediation of toxicants from the environment (Reid et al., 2019). Thus, human management, monitoring and mitigation of local environmental conditions like warming and nutrient run‐off, are crucial for the maintenance of urban pond ecosystems (Paerl & Otten, 2013; Peretyatko et al., 2009).
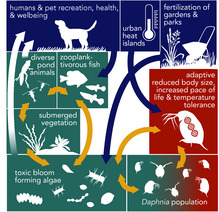

Most urban evolutionary biology research to date has focused on instances of nonadaptive evolution showing, for example, altered patterns of gene flow and genetic drift in cities (Bullock et al., 2018; Miles, Breitbart, et al., 2019; Schmidt et al., 2020). These genetic patterns can reflect human decisions to construct barriers and corridors that impact the dispersal and thus gene flow of both native and human‐affiliated species such as pests, disease vectors, and invasive species (Harris et al., 2016). In particular, overlaying genetic patterns on city maps has led to a more comprehensive understanding of dispersal and relatedness among populations of nuisance species (Combs, Puckett, Richardson, Mims, & Munshi‐South, 2018), and thus an ability to predict future spread of pest species and resistance alleles through neighbourhoods (Rost et al., 2009). There is evidence from genetic analyses of neutral genetic variation that native species are negatively affected by urban fragmentation (Delaney, Riley, & Fisher, 2010; Van Rossum, 2008), whereas exotic species can benefit from the deliberate transportation and establishment by humans who favour them for both private and public gardens and parks (Colla & MacIvor, 2017; Trusty, Goertzen, Zipperer, & Lockaby, 2007; Zengeya et al., 2017). Because human decisions and activities structure nearly every aspect of urban ecosystems, studying and quantifying their consequences and feedbacks will be essential for a holistic understanding of evolution in cities .

## 3 ECO‐EVOLUTIONARY DYNAMICS IN CITIES

The field of eco‐evolutionary dynamics emerged from growing evidence of the reciprocal feedbacks between ecological and evolutionary processes that are possible when both occur at similar temporal and spatial scales (Hairston, Ellner, Geber, Yoshida, & Fox, 2005; Hendry & Kinnison, 1999; Reznick & Ghalambor, 2001; Thompson, 1998). One of the central tenants of eco‐evolutionary dynamics is that evolutionary trait change within species (intraspecific variation) not only influences population dynamics (e.g. migration, reproduction), but also interactions between organisms and their surroundings, thereby affecting ecological patterns and processes like community composition and primary productivity (Des Roches et al., 2018; Fussmann et al., 2007; Hendry, 2017). Altered ecological conditions can then feed back to cause further evolutionary change. Feedbacks are at the centre of experiments and mathematical models of eco‐evolutionary dynamics, which have demonstrated their importance and prevalence in controlled laboratory settings as well as natural and altered habitats (Abrams & Matsuda, 1997; Bassar et al., 2010; Harmon et al., 2009; Loeuille & Leibold, 2008; Palkovacs & Post, 2009; Yoshida, Jones, Ellner, Fussmann, & Hairston, 2003). Many of these studies have underscored the importance of rapid evolution and genetic variation in conservation and management strategies for species impacted by anthropogenic threats (Allgeier et al., 2020; Merilä & Hendry, 2013; Nadeau & Urban, 2019; Urban et al., 2016; Wood, Palkovacs, & Kinnison, 2018; Lambert & Donihue, 2020). Still, relatively little research has explicitly examined the existence and role of eco‐evolutionary feedbacks in cities (but see Brans et al., 2017). Indeed, conservation in cities will benefit greatly from a better understanding of urban evolution and how it impacts management success (Lambert & Donihue, 2020).

Urban eco‐evolutionary feedbacks are particularly relevant because they have the potential to affect a great number of people through ecosystem and “evosystem” services (or “natures contributions to people”) and disservices (Bolund & Hunhammar, 1999; Jenerette, Harlan, Stefanov, & Martin, 2011; Pascual et al., 2014). These feedbacks, which can extend beyond the boundaries of cities themselves (Jiang, Deng, & Seto, 2013; Kaufmann et al., 2007; Seto et al., 2010), affect species persistence, abundance and population demographics, thereby influencing diverse ecological functions and both beneficial and detrimental ecosystem services (Faith et al., 2010). Further, eco‐evolutionary feedbacks towards humans can be unevenly distributed within and among cities leading to unequal distribution of services and disservices across human society (Bolund & Hunhammar, 1999; Jenerette et al., 2011; Pascual et al., 2014). For example, affluent neighbourhoods can have larger, more diverse (Jenerette et al., 2011; Oertli & Parris, 2019) and better interconnected green and blue spaces that support more abundant, genetically variable and therefore more stable populations of beneficial species such as pollinators (Gill et al., 2016). However, these neighbourhoods can also have a higher proportion of non‐native species in gardens and monoculture lawns that are manicured and eradicated of native weeds (Lerman & Warren, 2011; Tallamy, 2020). Green roofs, which are becoming a common feature of newer buildings, can be genetically depauperate and thus harmful to local conspecifics and pollinators unless careful consideration is given to the initial seed stock (Ksiazek‐Mikenas, Fant, & Skogen, 2019). Although non‐native species might initially boost diversity and ecosystem function (Wilson & Jamieson, 2019), they can become invasive through evolutionary processes such as hybridization (Culley & Hardiman, 2009; Rius & Darling, 2014) and introduce novel diseases and pests (Chifflet, Guzmán, Rey, Confalonieri, & Calcaterra, 2018; Eritja et al., 2005; Juliano & Philip Lounibos, 2005; Salyer, Bennett, & Buczkowski, 2014) that negatively affect native species (Godefroid, 2001; Shochat, Warren, Faeth, McIntyre, & Hope, 2006; Wania, Kühn, & Klotz, 2006).

Some of the most important eco‐evolutionary feedbacks on people living in cities occur through the spread of organisms and genes that provide “disservices” such as negative effects on human health and well‐being (Evans & Wellems, 2002). Again, the burdens of these detrimental feedbacks are unevenly distributed across the urban landscape. For example, rodenticide resistance in brown rats disproportionately affects the lower socio‐economic communities that are more burdened by these pests (Case Study Box 3a; Desvars‐Larrive et al., 2017). In some cases, humans have coevolved with urban pests such as mosquitos (Kamdem, Fouet, Gamez, & White, 2017; Sabeti et al., 2002) and their malaria‐causing pathogens (Case Study Box 3b; Evans & Wellems, 2002). Feedbacks from rapidly evolving pest and pathogen species may be particularly extreme in cities and neighbourhoods where human hosts are living in concentrated areas, such as in lower‐income public housing and apartment complexes (Booth et al., 2012; Byers, Lee, Patrick, & Himsworth, 2019; Combs et al., 2018; Koch et al., 2016; Saenz, Booth, Schal, & Vargo, 2012). For example, rampant urban bed bug infestations, again usually in lower‐income neighbourhoods, are an outcome of higher human density, frequent tenant and resident turnover, increased reliance on public transportation and the common exchange of second‐hand and used goods (Booth et al., 2012). Not only does increased turnover and human–human contact lead to more frequent colonization of these pests, but it also introduces adaptive alleles conferring resistance to common pesticides, thereby further facilitating their spread and persistence (Saenz et al., 2012). Similar transmission of resistance alleles has been documented in other pest and pathogen species such as head lice (Koch et al., 2016), German cockroaches (Wada‐Katsumata, Silverman, & Schal, 2013) and malaria (Kamdem et al., 2017). Higher connectivity in urban centres can in some cases promote genetic diversity and persistence in pest and pathogen populations by facilitating gene flow, such as with black widow spiders (Miles, Dyer, & Verrelli, 2018). Explicitly assessing the responses of organisms to features of urban ecosystems such as green space, pollution, waste and food availability will improve our understanding of the interface among social, ecological and evolutionary dynamics in cities.

Relatively little research has compared the strength of eco‐evolutionary feedbacks between urban and nonurban ecosystems (Miles, Breitbart, et al., 2019). In some cases, feedbacks might be magnified in urban areas: for example, white clover—a common herbaceous plant in urban and parks lawns—has adaptations that likely contribute to its continued persistence in lawns and parks (Case Study Box 3c; Johnson et al., 2018; Thompson et al., 2016), leading to positive feedbacks for beneficial species, such as pollinators and nitrogen‐fixing bacteria (Baude et al., 2016; Larson, Kesheimer, & Potter, 2014). Alternatively, feedbacks from evolutionary processes may be overshadowed or weakened due to external forces: for example, *Daphnia*—a genus of ubiquitous freshwater zooplankton—are known to exert strong top‐down control on algae and can adapt to increased temperatures in urban ponds (Case Study Box 3d; Brans, Jansen, et al., 2017). However, disturbances, such as extreme heat waves or extensive eutrophication following the build‐up of nutrient run‐off, can compromise *Daphnia*’s capacity to adapt and maintain its algae‐controlling ecological function. The loss of this function from the system can initiate drastic shifts in the pond ecosystem, including the spread of toxic algal blooms (Ger et al., 2016) that not only limit the diversity and abundance of insects, amphibians and submerged vegetation, but also present a public health concern to humans and their pets (Kosten et al., 2012; Thomaz & Cunha, 2010). Feedbacks from species like white clover and *Daphnia* may be more nuanced, though still broadly important for ecosystem function and services in cites.

## 4 TOWARDS AN URBAN SOCIO‐ECO‐EVOLUTIONARY FRAMEWORK

Despite an inherent spatial and temporal heterogeneity of cities, research on urban ecology and evolutionary biology often defaults to simplistic unidimensional, linear or dichotomous urban variables (e.g. urban versus nonurban, proportion of built‐up area and other land cover classes, human population density) that consider urbanization as a continuous gradient (McPhearson et al., 2016; Moll et al., 2019). Although these aggregate proxies are capable of capturing some urban variation, they often fail to encapsulate the complexity of urban systems that are driven by social and ecological interactions (Alberti et al., 2020; Schell et al., 2020). Acknowledging and incorporating spatial and temporal heterogeneity in these interactions will be important for studying urban eco‐evolutionary dynamics. For example, access to food, public transit routes, waste management and green space usually varies nonlinearly with urban zoning. Further, historical redlining practices that reflect underlying racist policies have led to an uneven distribution of infrastructure and social services that structure the urban ecosystem in many US cities (Schell et al. 2020; Grove et al., 2014; Locke et al., 2020; Roman et al., 2018). Below, we argue that study of socio‐eco‐evolutionary dynamics in cities requires an approach that addresses and acknowledges these complex, multivariate, and heterogeneous stressors. First, we describe how existing phenotypic and genomic approaches for studying eco‐evolutionary dynamics might be extended to include the social patterns and processes intrinsic to urban ecosystems. Second, we suggest how the coupled human and natural systems framework—a central tenant of urban ecology—might incorporate evolutionary biology, and by extension, eco‐evolutionary dynamics, to help understand socio‐ecological processes and feedbacks. Finally, we overview the opportunities for studying socio‐eco‐evolutionary dynamics, stressing a thorough and systematic identification of the demographic, cultural, political, economic and technological drivers that shape and are shaped by urban ecology and evolution.

### 4.1 Extending eco‐evolutionary dynamics to include human society

The concept of the evolving metacommunity (Definition: Box 1) is one example of a current framework in evolutionary ecology that can be used to study socio‐eco‐evolutionary dynamics in urban ecosystems. This framework considers organisms within networks of interconnected populations and communities (Urban & Skelly, 2006). Biological responses to environmental changes are therefore governed by a dynamic interplay between local and regional processes, including species sorting, adaptation, dispersal and gene flow (Urban & Skelly, 2006). Extending the evolving metacommunity theory to incorporate the effects of humans and social dimensions will be an important consideration in studying eco‐evolutionary dynamics in urban ecosystems. In these ecosystems, individuals, populations and communities are nested in a mosaic of habitats that are interconnected and fragmented by human activity and infrastructure. While roads, waterways and built structures isolate and restrict distribution in some species, they connect and disperse others that are more closely associated with humans (Miles, Rivkin, et al., 2019).

Humans might also be uniquely incorporated into evolving metacommunity models as species themselves. As with other interacting species, human populations are characterized by varying abundance and distribution that reflects their interactions with local environments. As important ecosystem engineers (Smith, 2007), humans can impose selection on other species. These other species and their adaptations might feed back to affect human densities, habitat choices, settlement and movement patterns. At broader spatial scales, urban influences on surrounding environments extend well beyond the geographic boundary of a city, making the hierarchical structure of the evolving metacommunity theory also helpful for studying urban eco‐evolutionary dynamics. Including social components like transportation infrastructure, neighbourhood cohesion, and socio‐economic geography may allow for more accurate predictions. For example, a consideration of international travel networks, national quarantine and customs policies, and trade embargos can help predict the evolution and spread of pathogenic, invasive and pest species (Helmus, Mahler, & Losos, 2014; Jones et al., 2008; Miles, Rivkin, et al., 2019). While challenging, a thorough incorporation of human social patterns and processes into ecological and evolutionary dynamics will lead to novel insights for understanding urban ecosystems.

### 4.2 Extending urban coupled human and natural systems to include evolution

An additional approach to studying socio‐eco‐evolutionary dynamics in cities is by extending urban ecology’s CHANS models (Box 2; Liu et al., 2007) to include evolutionary processes and feedbacks. These models have shown that human socio‐economic and demographic patterns and processes are reflected in infrastructure and other abiotic and biotic features of the urban ecosystem (Schaider, Swetschinski, Campbell, & Rudel, 2019; Tessum et al., 2019). Urban evolution research has simultaneously revealed that these same physical and biological characteristics can influence both the adaptive (Brans & De Meester, 2018; Whitehead et al., 2017) and nonadaptive (Combs et al., 2018; Munshi‐South, 2012) evolution of urban species. Indeed, recent work has shown that urban predictor variables that characterize socio‐economic heterogeneity, such as urban heat islands (Brans & De Meester, 2018) and environmental pollutants (Isaksson, 2015; Reid et al., 2016; Wirgin et al., 2011), can drive physiological and life‐history adaptations in organisms. Recent work in Baltimore, USA, has shown that tiger mosquitoes (*Aedes albopictus*) in low‐income neighbourhoods tend to have larger wing and body sizes—traits linked to increased fecundity, survival and ultimately spread of disease (Katz, Leisnham, & LaDeau, 2019). The distribution of these human influences is a direct result of socially driven urban form underpinned by exacerbating legacies of income inequality and segregation over decades and centuries (Grove et al., 2018; Roman et al., 2018). Integration of social processes and their relevant eco‐evolutionary feedbacks may therefore serve dual functions: first, by increasing our understanding of the value of ecological and evolutionary processes in cities, and second, by providing the applied tools to mitigate urban disturbances on ecosystems.

### 4.3 Opportunities for studying socio‐eco‐evolutionary dynamics

To fully understand urban eco‐evolutionary dynamics, we need to explicitly identify the mechanisms by which human society influences ecology, evolution and their feedbacks. Urban ecosystems are constantly changing as a result of social decisions and processes such as public policies and private landownership. Humans also interact dynamically within their communities through multiple networks like economic markets and public institutions. For example, urban residents depend on large‐scale built infrastructures (e.g. as electric power, water supply, food distribution and transportation networks) that sustain resource flows within and across cities (Childers et al., 2015). These interactions contribute to unique physical (e.g. sprawl), social (e.g. cultural and economic segregation) and economic (e.g. land values and use) properties of cities that can affect ecological and evolutionary processes on broad scales.

Urban ecosystems are subject to multiple drivers of human‐driven environmental change such that they often experience extreme climatic conditions across multiple axes. How different environmental conditions interact with one another and affect urban organisms is highly variable and poorly understood. Consequently, the responses of organisms to urbanization often cannot be predicted based on studies of any environmental condition in isolation. For example, researchers showed that bird life‐history traits were better predicted by a simple model that tested the effect of urban vs nonurban habitats compared to models that included four separate environmental variables that were each correlated with urbanization (temperature, humidity, artificial light and noise). The better fit of the simple model suggests that additional unmeasured variables account for the differences in life‐history along urban–rural gradients, and thus many ecological, social and evolutionary factors likely need to be included to accurately predict traits changes associated with urbanization (Sprau, Mouchet, & Dingemanse, 2017; Szulkin, Garroway, Corsini et al. 2020).

Landscape transformation, infrastructure development and complex social and political networks vary considerably across regions, causing heterogeneity within and among cities that can influence ecological and evolutionary processes (Alberti et al., 2020). For example, variation in land use patterns reflects a complex interplay among homeowners’ choices, real estate markets, local businesses and policymakers decisions (Alberti, 2008). These interactions can affect the arrangement and proportion of built and natural land cover, thereby influencing organisms and their habitats. Quantifying socio‐economic variables can help with the construction and parameterization of urban eco‐evolutionary dynamics models (McPhearson et al., 2016). These variables include the distribution of transportation networks (i.e. accessible from municipal resources), built infrastructure (i.e. from urban planning) and land use (i.e. from GIS and satellite imagery), as well as attributes of human demographics and society (i.e. from census and other survey data). Participatory science (also called citizen or community science) efforts in particular present an important opportunity both for collecting large‐scale eco‐evolutionary (Cooper, Dickinson, Phillips, & Bonney, 2007) and socio‐ecological data (Crain, Cooper, & Dickinson, 2014) and for promoting science to the general public using surveys, audiovisual data collection apps (e.g. SpiderSpotter, Bloomin’ Algae, iNaturalist, eBird, iSpot) and other technological platforms (Krasny, Russ, Tidball, & Elmqvist, 2014).

The relative predictability of urban sprawl also provides an important avenue for initiating longitudinal studies that collect baseline data and track the development and restoration of landscapes through time (Etterson et al., 2016). In particular, researchers can measure social, ecological and evolutionary parameters at pre‐, intermediate‐ and post‐urbanization time points and at different levels of biological organization, contrasting urbanized, urbanizing and nonurbanizing sites within and across cities. These research strategies can enable reconstruction of population genetic and phenotypic diversity and change, as well as community composition and species diversity over time. Socio‐demographic and socio‐economic changes can be monitored in parallel to determine potential drivers of eco‐evolutionary change in cities.

Identifying the underlying sources of phenotypic variation is crucial for assessing the relationships and feedbacks among social, ecological and evolutionary processes in urban ecosystems. Most traits are the product of both genetic and environmental factors. As a result, purely phenotypic studies can confound the inference of eco‐evolutionary dynamics if they do not account for the joint effects of plasticity and genetics on phenotypic variation and fitness (Brans, Jansen, et al., 2017; Govaert, Pantel, & De Meester, 2016; Perrier, Caizergues, & Charmantier, 2020). In particular, the inference of urban evolution in instances of polygenic inheritance necessitates standardized common garden or reciprocal transplant experiments to evaluate both the heritability and the fitness consequences of supposed urban adaptations (Thompson et al., 2016). For example, researchers used reciprocal transplants with common ragweed to identify local adaptation and divergent selection between populations in urban and nonurban habitats (Gorton, Moeller, & Tiffin, 2018). Studies like these can be replicated across multiple urban gradients and sampling plots within and among different cities and neighbourhoods to test the ubiquity and convergence of evolutionary trajectories (Santangelo, Miles, Breitbart, et al. 2020). Variance partitioning metrics (Govaert, 2018; Govaert et al., 2016; Lajoie & Vellend, 2015) can further help disentangle the relative contributions of plasticity and genetics underlying intraspecific trait variation, community ecology and ecosystem processes (Brans, Govaert, et al., 2017; Stoks, Govaert, Pauwels, Jansen, & Meester, 2016). Such analyses will be essential for understanding socio‐eco‐evolutionary dynamics.

## 5 LOOKING FORWARD: FUTURE STUDIES IN SOCIO‐ECO‐EVOLUTIONARY DYNAMICS

Urban ecosystems are fundamentally regulated, transformed and interconnected by human activity. Thus, integrating human social patterns and processes in urban evolution studies not only presents an opportunity for novel research, but is also imperative for accurately understanding contemporary ecological and evolutionary dynamics in cities. As we move forward, we argue that more fully integrating evolutionary ecology research with the social sciences to address socio‐eco‐evolutionary questions is critical because:

- Accurate predictions about urban coupled human and natural systems (CHANS) will require understanding the role of evolution in socio‐ecological systems over various timescales.
- A complete understanding of urban eco‐evolutionary dynamics will require an explicit consideration of social patterns and processes.
- The world is increasingly urbanized and the effects of cities extend beyond their borders. Hence, understanding ecological responses to global change will depend on our ability to address #1 & 2.

Studies of cities as coupled human and natural systems (CHANS) and of eco‐evolutionary dynamics have already provided insights into how urban ecosystems are likely to change over time. We now have the opportunity to leverage these existing bodies of work to create an integrative framework that more fully resembles the simultaneous social, ecological and evolutionary dynamics in urban ecosystems. We encourage a new collaboration among social scientists, ecologists and evolutionary biologists to develop more sophisticated questions and increasingly accurate models of urban systems, and garner a greater understanding of dynamics both within and beyond city boundaries. Understanding urban evolutionary biology will have vast implications for socio‐ecological policies such as those relating to biodiversity management and ecological restoration as well as human health, well‐being and equity. Additionally, we suggest specific, important and timely questions that can be addressed with an integrated socio‐eco‐evolutionary framework (Questions: Box 4).

BOX 4 **Outstanding questions that could be addressed using a socio‐eco‐evolutionary framework**

Integrating insights from social sciences, ecology and evolutionary biology can help us address critical questions about urban systems. This understanding will feed back to improve our knowledge and predictions about how ecosystems respond to global change. Here, we propose ten key questions to inform an integrated socio‐eco‐evolutionary framework.

1. How can incorporating methods from the social sciences improve our understanding of eco‐evolutionary dynamics?

2. How do socio‐eco‐evolutionary dynamics scale with the spatial redistribution and generation lengths of humans and associated organisms across space and time?

3. What is the relevance and magnitude of evolutionary feedbacks to ecological and social patterns and processes in different urban contexts?

4. Can we predict the ways that interspecific interactions will influence eco‐evolutionary dynamics in cities and the ways in which social drivers will modify these dynamics and patterns?

5. How important are local dynamics and species identities to eco‐evolutionary dynamics in cities? What are the components of a cohesive theory that is relevant to all or most urban systems, and when do local ecology, culture and politics idiosyncratically shape outcomes?

6. How can eco‐evolutionary dynamics feed back to influence social processes in cities? In what ways can social systems change in response to evolutionary changes that are induced by urbanization?

7. How can this multidimensional framework help us better understand the resilience of urban ecosystems to pulse disturbances, such as extreme weather events, and ramping disturbances, such as climate change?

8. What elements of human social constructs (e.g. socio‐economic, cultural, religious, philosophical, political and aesthetic) are likely to impact socio‐eco‐evolutionary dynamics?

9. Under what circumstances are eco‐evolutionary processes stronger or weaker in urban compared to nonurban areas?

10. How do socio‐eco‐evolutionary changes in cities affect the influences of cities on surrounding landscapes?

Cities provide exciting systems to expand our knowledge of eco‐evolutionary dynamics and their social causes and consequences. Studying the social dimensions of eco‐evolutionary dynamics in cities will improve our understanding of the complexity of urban biological communities, which will be increasingly crucial for conserving and maximizing ecosystem functions and contributions to people within and outside cities. Research on urban socio‐eco‐evolutionary dynamics provides a unique opportunity to study evolving metacommunities, the interplay between local and regional responses, and the presence and strength of eco‐evolutionary feedbacks across multiple taxonomic groups. Just as urban ecology grew to consider the social complexity of cities and eco‐evolutionary dynamics integrated the rapid pace of evolution, socio‐eco‐evolutionary research must recognize the dynamism resulting from the interplay of social, ecological and evolutionary dimensions within urban systems.

## CONFLICT OF INTEREST

None declared.
